# Comparative efficacy of interpersonal psychotherapy and antidepressant medication for adult depression: a systematic review and individual participant data meta-analysis

**DOI:** 10.1017/S0033291724001788

**Published:** 2024-10

**Authors:** Zachary D. Cohen, Jasmijn Breunese, John C. Markowitz, Erica S. Weitz, Steven D. Hollon, Dillon T. Browne, Paola Rucci, Carolina Corda, Marco Menchetti, Myrna M. Weissman, R. Michael Bagby, Lena C. Quilty, Marc B. J. Blom, Mario Altamura, Ingo Zobel, Elisabeth Schramm, Carlos Gois, Jos W. R. Twisk, Frederik J. Wienicke, Pim Cuijpers, Ellen Driessen

**Affiliations:** 1 Department of Psychology, University of Arizona, Tucson, AZ, USA; 2 Department of Clinical Psychology, Behavioural Science Institute, Radboud University, Nijmegen, Netherlands; 3 Department of Psychiatry, Columbia University Vagelos College of Physicians and Surgeons, New York, NY, USA; 4 New York State Psychiatric Institute, New York, NY, USA; 5 Department of Psychiatry, University of Pennsylvania, Philadelphia, PA, USA; 6 Department of Psychology, Vanderbilt University, Nashville, TN, USA; 7 Department of Psychology, University of Waterloo, Waterloo, ON, Canada; 8 Department of Biomedical and Neuromotor Sciences, University of Bologna, Bologna, Italy; 9 Department of Experimental, Diagnostic and Specialty Medicine, University of Bologna, Bologna, Italy; 10 Departments of Psychology and Psychiatry, and Graduate Department of Psychological Clinical Science, University of Toronto Scarborough, Scarborough, ON, Canada; 11 Centre for Addiction and Mental Health and Department of Psychiatry, Campbell Family Mental Health Research Institute, University of Toronto, Toronto, ON, Canada; 12 Parnassia Groep, Den Haag, Netherlands; 13 Department of Clinical and Experimental Medicine, University of Foggia, Foggia, Italy; 14 Psychology School, Hochschule Fresenius, University of Applied Sciences Berlin, Berlin, Germany; 15 Department of Psychiatry and Psychotherapy, Medical Center, Faculty of Medicine, University of Freiburg, Freiburg im Breisgau, Germany; 16 Department of Psychiatry, University of Lisbon, Lisbon, Portugal; 17 Department of Epidemiology and Data Science, Amsterdam University Medical Centers, Amsterdam, Netherlands; 18 Department of Clinical, Neuro and Developmental Psychology, Amsterdam Public Health research institute, Vrije Universiteit Amsterdam, Amsterdam, Netherlands; 19 International Institute for Psychotherapy, Babeș-Bolyai University, Cluj-Napoca, Romania; 20 Depression Expertise Center, Pro Persona Mental Health Care, Nijmegen, Netherlands

**Keywords:** Depression, antidepressant medication, interpersonal psychotherapy, efficacy, individual participant data meta-analysis

## Abstract

Interpersonal psychotherapy (IPT) and antidepressant medications are both first-line interventions for adult depression, but their relative efficacy in the long term and on outcome measures other than depressive symptomatology is unknown. Individual participant data (IPD) meta-analyses can provide more precise effect estimates than conventional meta-analyses. This IPD meta-analysis compared the efficacy of IPT and antidepressants on various outcomes at post-treatment and follow-up (PROSPERO: CRD42020219891). A systematic literature search conducted May 1st, 2023 identified randomized trials comparing IPT and antidepressants in acute-phase treatment of adults with depression. Anonymized IPD were requested and analyzed using mixed-effects models. The prespecified primary outcome was post-treatment depression symptom severity. Secondary outcomes were all post-treatment and follow-up measures assessed in at least two studies. IPD were obtained from 9 of 15 studies identified (*N* = 1536/1948, 78.9%). No significant comparative treatment effects were found on post-treatment measures of depression (*d* = 0.088, *p* = 0.103, N = 1530) and social functioning (*d* = 0.026, *p* = 0.624, *N* = 1213). In smaller samples, antidepressants performed slightly better than IPT on post-treatment measures of general psychopathology (*d* = 0.276, *p* = 0.023, *N* = 307) and dysfunctional attitudes (*d* = 0.249, *p* = 0.029, *N* = 231), but not on any other secondary outcomes, nor at follow-up. This IPD meta-analysis is the first to examine the acute and longer-term efficacy of IPT *v*. antidepressants on a broad range of outcomes. Depression treatment trials should routinely include multiple outcome measures and follow-up assessments.

## Introduction

Depression is a highly prevalent and debilitating psychiatric disorder. Its status as the single largest cause of global disability underscores the need for effective and efficient treatment options (World Health Organization, 2017). Current practice guidelines recommend antidepressant medications and evidence-based psychotherapies as first-line treatments for depressive disorders (American Psychological Association, 2019; National Institute for Health and Care Excellence, 2022). Although antidepressant medications are widely available, effective, and represent a commonly accessed form of treatment (Cipriani et al., 2018), issues surrounding long-term use (Bet, Hugtenburg, Penninx, & Hoogendijk, 2013), side effects (Ferguson, 2001), and patients' preference for psychotherapy (McHugh, Whitton, Peckham, Welge, & Otto, 2013; van Schaik et al., 2004) highlight the importance of alternative approaches. Moreover, antidepressants may have limited benefit in situations where social context plays a role (Cuijpers et al., 2011). One alternative is interpersonal psychotherapy (IPT), a time-limited, evidence-based, present-focused psychological intervention that emphasizes the role of negative relational and interpersonal experiences in the onset and course of depression (Weissman, Markowitz, & Klerman, 2018). IPT addresses disruptive social relationships and interpersonal behavioral patterns to improve social functioning, skills, and support (Weissman, Klerman, Prusoff, Sholomskas, & Padian, 1981), and thereby to ameliorate depression (Markowitz & Weissman, 2012). This therapeutic approach, therefore, may particularly suit individuals with depression experiencing stressful life events such as loss, separation, interpersonal disputes, and other major life changes (Markowitz & Weissman, 2004, 2012).

Widespread empirical evidence supports the efficacy of both IPT and antidepressant medications in the acute phase treatment of adult depression (Cipriani et al., 2018; de Mello, de Jesus Mari, Bacaltchuk, Verdeli, & Neugebauer, 2005). In comparing their treatment effects, some conventional meta-analyses (which rely on study-level data extracted from publications) found no significant differences (Cuijpers, Donker, Weissman, Ravitz, & Cristea, 2016; de Mello et al., 2005), whereas one reported small effect sizes favoring antidepressants (Cuijpers et al., 2011). The aggregation of study-level data from publications limits conventional meta-analyses, however, making them dependent on the quality of published results and prone to bias (Riley, Lambert, & Abo-Zaid, 2010; Stewart & Parmar, 1993). Moreover, previous meta-analyses comparing IPT and antidepressants tend to narrowly focus on treatment acceptability and depressive symptom severity at treatment completion. As the depressive syndrome extends beyond depressive symptomatology (Cuijpers, 2020), several authors have emphasized the importance of patients' physical, social, and psychological condition as outcomes (Cuijpers, 2020; Hirschfeld et al., 2000; Kennedy, Eisfeld, & Cooke, 2001). Moreover, long-term treatment effects are important to consider given the highly recurrent nature of depression. Although a recent network meta-analysis reported some evidence for an advantage of psychotherapies over medications in sustained response after one year (Furukawa et al., 2021), the comparative enduring effects of IPT and antidepressants are currently unclear.

This study addresses the aforementioned limitations by using an individual participant data (IPD) meta-analytic approach to compare the efficacy of IPT and antidepressants at the end of acute treatment (post-treatment) and at long-term follow-up on a broad range of outcome measures, including depressive symptom severity, social functioning, and measures of well-being (e.g. quality of life and health status; Driessen et al., 2021). IPD meta-analyses aggregate participant-level data from multiple clinical trials and can contribute considerably to the existing literature (Cuijpers et al., 2022). Raw participant data can access outcome variables that published articles may not have reported (Riley et al., 2010; Stewart & Parmar, 1993). Moreover, IPD enables standardization of analytic approaches across studies (Riley et al., 2010), and its independence from how results are reported in published articles can improve reliability of results (Riley et al., 2010; Stewart & Parmar, 1993). Although IPD meta-analyses have been conducted comparing the efficacy of antidepressants with cognitive behavioral therapies (CBT; Furukawa et al., 2018; Weitz et al., 2015), no IPD meta-analyses have compared the efficacy of antidepressants with a non-cognitive behavioral psychotherapy (Driessen et al., 2021).

## Methods

### Information sources, search strategy, and selection process

The study was registered (PROSPERO: CRD42020219891) and its detailed protocol was published (Driessen et al., 2021). Divergences from the protocol consisted of analyses precluded by unavailable data (see online Supporting information in the Appendix). Studies were identified by searching the METAPSY database of randomized clinical trials (RCTs) examining the efficacy of psychological treatments for depression (www.metapsy.org). This database was created through comprehensive literature searches in PubMed, PsycINFO, Embase, and the Cochrane Library until May 1st, 2023. The exact search terms can be retrieved from https://osf.io/nv3ea/. Two raters independently assessed all references and relevant full-text papers (Driessen et al., 2021). Disagreements were resolved through consensus. In addition, reference lists of prior reviews were inspected, and the International Society of Interpersonal Psychotherapy listserv was contacted for missed studies. There were no restrictions concerning the years in which a study was conducted, or regarding language, date, or status of publication (i.e. unpublished, published/in press).

### Eligibility criteria

Studies were included if they were RCTs comparing IPT and antidepressants in the acute phase treatment of adults with depression. IPT had to conform to the manuals developed by Klerman and Weissman (Klerman, Weissman, Rounsaville, & Chevron, 1984; Weissman et al., 2018) or to the manual for the shortened version, interpersonal counseling (IPC; Weissman et al., 2014). Sessions could be delivered in any format, setting, or time frame as long as a clinician provided the therapy. Any oral antidepressant medication within the therapeutic dose range was included: e.g. selective serotonin reuptake inhibitors (SSRIs), tricyclic antidepressants (TCAs), and monoamine oxidase inhibitors. Patients had to be 18 years or older and were considered depressed if they met specified criteria (e.g. Diagnostic and Statistical Manual of Mental Disorders [DSM]) for major depressive disorder or another unipolar mood disorder assessed by means of a semi-structured interview or clinicians' assessment, or if they presented a score at or above a validated cut-off indicating the likelihood of clinically significant depressive symptoms on an evaluator-assessed, clinician-assessed, or self-reported measure of depression. Comorbid mental and somatic disorders were allowed.

### Data collection

Authors of the included studies were contacted according to a multi-step protocol (Driessen et al., 2021) and invited to contribute their anonymized patient-level data. The request embraced all outcome measures assessed in the study. Received data were checked against the published articles for accuracy and completeness (Driessen et al., 2021), and for invalid, out-of-range and inconsistent items. Discrepancies were discussed with the authors. All outcome measures and assessment points were listed for each study. Only data from relevant phases and comparisons were used for analysis (e.g. only outcomes measured before augmentation following non-response to monotherapy). Prespecified study characteristics, treatment characteristics, IPT/antidepressant quality characteristics, and effect size data were extracted from the published articles (Driessen et al., 2021).

### Measures

The prespecified primary outcome was depressive symptom severity at the end of acute treatment, defined as the primary continuous measure of depression at post-treatment according to the component study authors (Driessen et al., 2021). Secondary outcomes included depressive symptom severity at follow-up and all outcome measures other than depression (e.g. quality of life, social functioning, health status) assessed at post-treatment or follow-up in at least two studies (Driessen et al., 2021). Because individual studies used different instruments to assess outcomes (for an overview see Appendix Table 1), scores were standardized by conversion into *z-*scores within time-point and study (Driessen et al., 2021).

### Bias assessments

Two independent raters assessed risk-of-bias in the included studies at the outcome level using the Cochrane Collaboration's risk-of-bias tool for randomized trials (version 2; Higgins, Savović, Page, Elbers, & Sterne, 2022). Disagreements were resolved by consensus or discussed with a third rater. Publication bias was evaluated by assessing asymmetry in funnel plots and Egger's test of the intercept for meta-analyses including 10 or more studies (Sterne et al., 2011). Data-availability bias was examined by comparing studies that contributed IPD with those that did not regarding study characteristics (χ^2^-analyses in SPSS Statistics, version 28.0) and effect sizes on depression outcomes based on publication-extracted data (conventional meta-analysis subgroup analysis in Comprehensive Meta-analysis, version 3.3.070).

### IPD meta-analyses

One-stage IPD meta-analyses were conducted using mixed models with restricted maximum-likelihood estimation in MLwiN (version 2.35; Burke, Ensor, & Riley, 2017). Analyses were based on intention-to-treat samples insofar as possible. Follow-up data were excluded from post-treatment analyses as additional help-seeking could not be controlled for.

Following recommendations by Twisk et al. (2018), the basic model included a main effect for time (categorical, represented by dummy variables), a time-by-treatment interaction, a random intercept for study (to account for the clustering of participants within studies), a random intercept for participants (to account for clustering of repeated measures within participants), and fixed slopes. In this model, the time-by-treatment interaction's regression coefficient indicates the comparative treatment effect. For analyses with >2 time points, log-likelihood change was evaluated to decide whether to add a random slope for the time-by-treatment interaction at the study level (to account for different treatment effects between studies).

Multiple prespecified sensitivity analyses were conducted to examine the robustness of the findings. To examine the impact of risk-of-bias and treatment quality, corresponding items were added as covariates to the mixed-effect models. Analyses were repeated for the subset of studies that scored positive on all treatment quality items. Sensitivity analyses were conducted using unstandardized scores for each specific outcome instrument assessed in two or more studies. Sensitivity analyses were conducted within the subset of studies with post-treatment outcomes between 4 and 8 weeks and between 12 and 20 weeks. To examine potential outcome differences, analyses were also repeated in the following subgroups of studies; IPT only *v.* IPC, dysthymia *v.* other unipolar mood disorders, primary *v.* secondary/tertiary care, adults *v.* older adults, and SSRIs *v.* TCAs or nefazodone. Sensitivity analyses including two or more studies are described here (all are reported in the Appendix). Finally, we explored response rates in the observed data, defining response as ⩾50% symptom reduction from pre- to post-treatment on the primary continuous depression measure according to the component study authors.

## Results

### Included studies

**Figure 1.**
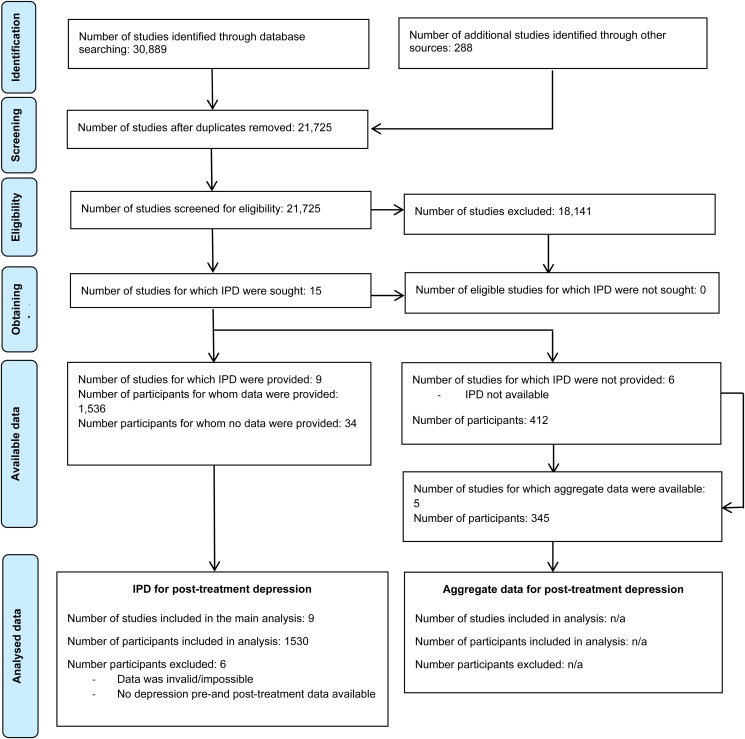
PRISMA IPD flow diagram.

Figure 1 presents the PRISMA flow diagram. The systematic literature search yielded 15 studies meeting eligibility criteria (*N* = 1948; for references see Appendix Table 2). IPD were obtained for 9 studies, comprising 1536 (78.9%) patients (IPT: *N* = 770; 50.1%, antidepressants: *N* = 766; 49.9%). Table 1 summarizes study characteristics. Depression inclusion criteria primarily consisted of a DSM diagnosis of major depressive disorder in combination with a Hamilton Depression Rating Scale (HAM-D) score indicating the likelihood of clinically significant depressive symptoms. One study investigated patients with dysthymic disorder. Seven of nine studies focused on depressed adults in general, while two studies targeted a specific group: post-stroke depression and type 2 diabetes mellitus. Seven studies examined IPT ranging from 8 to 20 sessions, whereas two examined IPC (6 to 8 sessions). All but one study applied an individual treatment format. Antidepressants were mainly SSRIs. Three studies conducted naturalistic follow-up assessments ranging from approximately 6 months to 2 years post-baseline. No additional IPT sessions were provided, but in one study patients assigned to antidepressants were offered medication throughout the entire 2-year follow-up period. Patients self-reported gender was mostly female (*N* = 1079/1536; 70.2%) with a mean age of 42.6 years (*SD* = 13.4, *N* = 1488).

**Table 1.** Characteristics of included studies

Study	*N* (ADM/IPT)^a^	Country	Recruitment	Target group	Depression diagnosis	ADM type	IPT format	*N*_sessions_	Follow-up
IPD available									
Altamura et al. (2017)	55 (28/27)	Italy	Clinical	Adults	Major depressive disorder (DSM-5); HAM-D ⩾ 8	SSRI	Individual^b^	6	
Blom et al. (2007)	97 (47/50)	Netherlands	Clinical	Adults	Major depressive disorder (DSM-4); HAM-D ⩾ 14	Other	Individual	12	
Browne et al. (2002)	460 (229/231)	Canada	Clinical and community	Adults	Dysthymic disorder, with or without major depressive disorder (DSM-4)	SSRI	Individual	12	1 and 2^c^ years
Elkin et al. (1989)	126 (63/63)	USA	Clinical	Adults	Major depressive disorder (RDC); HAM-D ⩾ 14	TCA	Individual	16–20	6, 12, and 18^c^ months
Finkenzeller, Zobel, Rietz, Schramm, and Berger (2009)	51 (24/27)	Germany	Other	Adults with post-stroke depression	Depressive disorder (ICD-10); HADS > 7; HAM-D ⩾ 14	SSRI	Group	8–16	
Frank et al. (2011)	291 (142/149)	USA; Italy	Clinical and community	Adults	Major depressive episode (DSM-4); HAM-D ⩾ 15	SSRI	Individual	12	
Gois et al. (2014)	34 (17/17)	Portugal	Other	Adults with type 2 diabetes mellitus	Major depression (DSM-4); HADS ⩾ 7; MADRS ⩾ 17	SSRI	Individual	12	
Menchetti et al. (2014)	287 (144/143)	Italy	Clinical	Adults	Major depressive episode (DSM-4); HAM-D ⩾ 13	SSRI	Individual^b^	6–8	
Quilty, McBride, and Bagby (2008)	135 (72/63)	Canada	Clinical and community	Adults	Major depressive disorder (DSM-4)	Other	Individual	16–20	Variable length (27–80 weeks)^c^
No IPD available									
Markowitz, Kocsis, Bleiberg, Christos, and Sacks (2005)	47 (24/23)	USA	Clinical and community	Adults	Dysthymic disorder with early onset (DSM-4); HAM-D > 13; GAF < 61	SSRI	Individual	16–18	
Martin, Martin, Rai, Richardson, and Royall (2001)	28 (15/13)	UK	Clinical	Adults	Major depressive episode (DSM-4); HAM-D ⩾ 18	SSRI	Individual	6	
O'Hara et al. (2019)	67 (33/34)	USA	Other	Women with postpartum depression	Major depressive episode (DSM-4); HAM-D ⩾ 15	SSRI	Individual	12	
Schulberg et al. (1996)	184 (91/93)	USA	Clinical	Adults	Major depression (DSM-3-R); HAM-D ⩾ 13	TCA	Individual	16	
Sloane, Staples, and Schneider (1985)	37 (18/29)	USA	Other	Older adults	Major depressive disorder (RDC); HAM-D ⩾ 17	TCA	Individual	6	
Weissman et al. (1979)	49 (24/25)	USA	Clinical	Adults	Major depression (SADS; RDC); Raskin Three Area Depression Scale ⩾ 7	TCA	Individual	16	

*Note.* ADM, antidepressant medication; DSM, Diagnostic and Statistical Manual of Mental Disorders; GAF, Global Assessment of Functioning; HADS, Hospital Anxiety and Depression Scale; HAM-D, Hamilton Depression Rating Scale; ICD-10, International Statistical Classification of Diseases and Related Health Problems, 10th edition; IPD, individual participant data; IPT, interpersonal psychotherapy; MADRS, Montgomery Åsberg Depression Rating Scale; *N*, number of patients; *N*_sessions_, number of IPT or IPC sessions; RDC, Research Diagnostic Criteria; SADS, Schedule for Affective Disorders and Schizophrenia; SSRI, selective serotonin reuptake inhibitor; TCA, tricyclic antidepressant.

a Number of patients receiving IPT or ADM monotherapy.

b Therapy format included IPC.

c Used as primary follow-up assessment point in the analyses.

### Bias assessments

**Figure 2.**
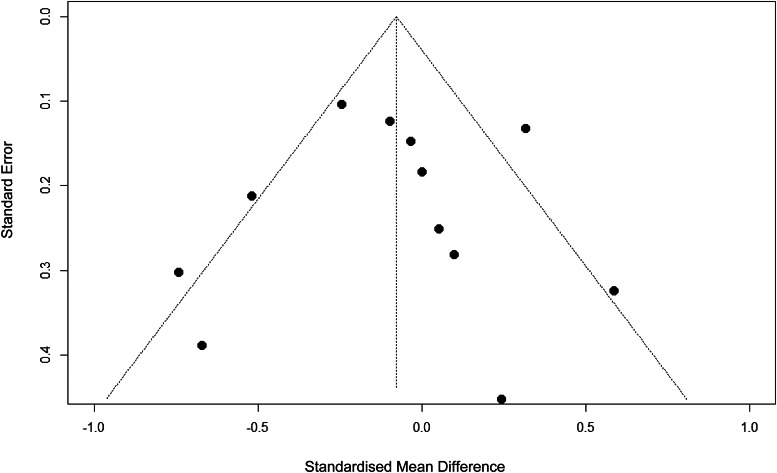
Funnel plot of effect estimates of studies examining IPT and antidepressants for depression.

Analyses found no significant differences in depression outcome effect sizes (*Q* = 1.434, *p* = 0.231) between studies for which IPD were (*d* = −0.010, 95% confidence interval [CI] [−0.230 to 0.210]) and were not available (*d* = −0.278, 95% CI [−0.658 to 0.101]). Studies for which IPD were obtained were more likely to be conducted in Europe than in the United States (χ^2^(4, *N* = 15) = 9.377, *p* = 0.025), but no differences were found in recruitment, target group, or depression criteria (*p*s > 0.12; Appendix Table 3). The funnel plot showed some asymmetry (Fig. 2), but Egger's test was not significant (*b* = −0.097, *SE* = 1.183, *p* = 0.936).

Table 2 presents the risk-of-bias assessment. For each outcome measure, overall risk-of-bias was determined to be either ‘high’ or ‘of concern’. The three main sources of risk-of-bias were studies' lack of an available prespecified research plan, reliance on outcomes assessed by self-report instrument, and failure to retain a full intention-to-treat sample.

**Table 2.** Risk-of-bias per outcome variable of included studies for which IPD were obtained

Outcome domain	Study	Randomization process	Deviations from the intended interventions	Missing outcome data	Measurement of the outcome	Selection of the reported results	Overall
Depression	Altamura, 2017	+	+	+	+	+/−	+/−
	Blom, 2007	+	+	−	+	+/−	+/−
	Browne, 2002	+	+	+	+	+/−	+/−
	Elkin, 1989	+	+/−	+	+	+/−	+/−
	Finkenzeller, 2009	+	+	+	+	+/−	+/−
	Frank, 2011	+	+	−	+	+/−	+/−
	Gois, 2014	+	+	+	+	+/−	+/−
	Menchetti, 2014	+	+/−	+	+	+	+/−
	Quilty, 2008	+/−	+	+	−	+/−	−
Anxiety	Altamura, 2017	+	+	+	+	+/−	+/−
	Finkenzeller, 2009	+	+	+	−	+/−	−
General psychopathology	Blom, 2007	+	+	−	+/−	+/−	+/−
	Elkin, 1989	+	+/−	+	−	+/−	−
	Quilty, 2008	+/−	+	−	−	+/−	−
Quality of life	Frank, 2011	+	+	−	−	+/−	−
	Menchetti, 2014	+	+/−	+	−	+/−	−
Social functioning	Blom, 2007	+	+	−	−	+/−	−
	Browne, 2002	+	+	+	−	+/−	−
	Elkin, 1989	+	+/−	+	+	+/−	+/−
	Frank, 2011	+	+	−	−	+/−	−
	Menchetti, 2014	+	+/−	+	−	+/−	−
Interpersonal problems	Blom, 2007	+	+	−	−	+/−	−
	Quilty, 2008	+/−	+	−	−	+/−	−
Coping mechanisms	Blom, 2007	+	+	−	−	+/−	−
	Browne, 2002	+	+	+	−	+/−	−
Personality traits	Blom, 2007	+	+	−	−	+/−	−
	Quilty, 2008	+/−	+	+	−	+/−	−
Dysfunctional attitudes	Elkin, 1989	+	+/−	+	−	+/−	−
	Quilty, 2008	+/−	+	+	−	+/−	−

*Note.* +, low risk-of-bias; +/−, some concerns; −, high risk-of-bias.

### IPD meta-analyses

Table 3 summarizes comparative treatment effects for all outcome measures at post-treatment and follow-up (for all sensitivity analyses see Appendix Table 4). There was no significant difference in treatment effect between IPT and antidepressants on post-treatment measures of depression (*d* = 0.088, 95% CI [−0.018 to 0.194], *p* = 0.103, *k* = 9, *N* = 1530). Sensitivity analyses indicated significant depression treatment effects favoring antidepressants when only including studies examining IPT (*d* = 0.170, 95% CI [0.048–0.292], *p* = 0.006, *k* = 7, *N* = 1189), using the Montgomery Åsberg Depression Rating Scale as outcome (*b* = 2.413, 95% CI [0.835–3.991], *p* = 0.003, *k* = 2, *N* = 494), or with high treatment quality (*d* = 0.139, 95% CI [0.021–0.257], *p* = 0.021, *k* = 5, *N* = 1260). All other sensitivity analyses including two or more studies indicated non-significant differences in treatment effect (Appendix Table 4). Based on observed data, 49.5% (*N* = 310/626) of the participants in the IPT conditions and 55.2% (*N* = 341/618) in the antidepressant conditions met the response criterion at the post-treatment assessment.

**Table 3.** Comparative treatment effects of IPT and antidepressants at post-treatment and follow-up

				95% CI		
Outcome	*k*	*N*	*d*^a^	Lower	Upper	*p*
Post-treatment						
Depression	9	1530	0.088	−0.018	0.194	0.103
Social functioning	5	1213	0.026	−0.078	0.130	0.624
General psychopathology	3	307	0.276	0.039	0.513	0.023*
Quality of life	2	567	−0.049	−0.198	0.100	0.519
Coping – problem solving	2	513	−0.059	−0.226	0.108	0.488
Coping – avoidance	2	509	0.041	−0.118	0.200	0.613
Dysfunctional attitudes	2	231	0.249	0.026	0.472	0.029*
Personality – neuroticism	2	192	0.102	−0.188	0.392	0.491
Personality – extraversion	2	192	−0.054	−0.293	0.185	0.658
Personality – agreeableness	2	192	0.169	−0.113	0.451	0.241
Personality – conscientiousness	2	192	−0.050	−0.297	0.197	0.691
Personality – openness	2	191	0.133	−0.110	0.376	0.283
Interpersonal problems	2	190	−0.038	−0.263	0.187	0.741
Anxiety	2	106	−0.200	−0.555	0.155	0.269
Follow-up						
Depression	3	716	0.150	−0.023	0.323	0.088
Social functioning	2	550	−0.014	−0.179	0.151	0.868
General psychopathology	2	226	−0.003	−0.309	0.303	0.985

*k*, number of studies; *N*, number of patients; *d*, Cohen's *d* effect size.

a Positive effect sizes indicate an advantage of antidepressants over IPT, except for the quality of life outcome measure where a negative effect size indicates an advantage of antidepressants over IPT.

* Statistical significance (*p* < 0.05).

At follow-up, no significant differences in treatment effects were found on depression measures (*d* = 0.150, 95% CI [−0.023 to 0.323], *p* = 0.088, *k* = 3, *N* = 716). Small but significant treatment effect differences favoring antidepressants were found on post-treatment measures of general psychopathology (*d* = 0.276, 95% CI [0.039–0.513], *p* = 0.023, *k* = 3, *N* = 307) and dysfunctional attitudes (*d* = 0.249, 95% CI [0.026–0.472], *p* = 0.029, *k* = 2, *N* = 231), but not on post-treatment measures of social functioning, quality of life, coping, personality, interpersonal problems, and anxiety, nor for social functioning and general psychopathology at follow-up (Table 3). Sensitivity analyses replicated the significant depression treatment effect favoring antidepressants on post-treatment measures of dysfunctional attitude using unstandardized scores (*b* = 8.550, 95% CI [1.110–15.990], *p* = 0.024, *k* = 2, *N* = 231). All other sensitivity analyses including two or more studies indicated non-significant differences in treatment effect (Appendix Table 4). The proportion of total variability due to between-study heterogeneity (*I*^2^) did not exceed 8% across any analysis.

## Discussion

This IPD meta-analysis examined the comparative efficacy of IPT and antidepressant medication as acute phase treatments for adult depression. No significant difference was found between IPT and antidepressants for treatment effect on depressive symptoms at post-treatment, the primary outcome. Antidepressants were found to be more efficacious than IPT in the sensitivity analyses that excluded studies examining IPC and that excluded studies with low treatment quality ratings, but the effect size estimates (respectively, *d* = 0.17 and *d* = 0.14) fell below the threshold of a clinically significant effect (*d* ⩾ 0.24; Cuijpers, Turner, Koole, Van Dijke, & Smit, 2014). These findings align with prior conventional meta-analyses comparing IPT and antidepressants that found no significant differences or only small effect sizes favoring antidepressants (Cuijpers et al., 2011, 2016; de Mello et al., 2005). They are also consistent with an IPD meta-analysis of antidepressants *v.* CBT, another leading evidence-based psychotherapy for depression, which reported a small but significant superiority of antidepressants on post-treatment HAM-D and a non-significant trend on self-reported depressive symptoms (Weitz et al., 2015).

Despite the recurrent nature of depression, comparative follow-up effects for treatments have received limited attention in the literature. Addressing this key question, this study considered longer term comparative treatment effects of IPT and antidepressants and found no significant differences on follow-up measures of depression. This finding contrasts with a prior meta-analysis suggesting that acute phase psychotherapy may have superior long-term benefits compared to acute phase antidepressant medication treatment (Imel, Malterer, McKay, & Wampold, 2008). We consider the current finding preliminary as it was based only on three studies, in one of which antidepressants continued to be offered throughout the entire 2-year follow-up period whereas no additional IPT was provided (Browne et al., 2002).

The focus of prior conventional meta-analyses on depressive symptom outcomes is problematic as depression is known to affect a broad spectrum of functional areas (Cuijpers, 2020). This study advances the literature by including a wide range of additional outcome domains: anxiety, quality of life, general psychopathology, social functioning, interpersonal problems, dysfunctional attitudes, personality, and coping mechanisms. An early study by Weissman et al. (1981) found that patients treated with IPT experienced greater improvement in their social functioning relative to patients treated with medication after one year, though not acutely. Although we could not examine social functioning at 1-year follow-up, the large sample size afforded by the IPD approach (*N* = 1213) supports the reliability of our null finding regarding between-treatment differences in social functioning at post-treatment.

Moreover, albeit based on smaller samples, comparative effect sizes for quality of life and coping at post-treatment, and for social functioning at follow-up were very small, with 95% CIs not exceeding the border of what is considered a clinically significant effect (Cuijpers et al., 2014). Findings regarding personality, interpersonal problems, and anxiety at post-treatment, and general psychopathology at follow-up, should be considered preliminary given the small sample sizes and wide CIs. Antidepressant medications did perform slightly better than IPT on post-treatment measures of general psychopathology and dysfunctional attitudes. Future work should seek to replicate these results in larger samples.

### Strengths and limitations

This IPD meta-analysis examined the acute and longer-term comparative efficacy of IPT and antidepressants across a broad range of outcome domains. The IPD meta-analytic approach allowed the inclusion of outcomes not previously reported. Beyond depression symptom severity, this study examined eight additional outcomes, providing a broader perspective on the effects of antidepressant treatments (Cuijpers, 2020). Working with IPD allowed conduct of intention-to-treat analyses for most of the included studies, and standardization of analytic methods across studies, thus reducing bias and yielding more reliable estimated effect sizes than conventional meta-analyses (Stewart & Parmar, 1993). The included studies were generally comparable in their target group, depression inclusion criteria, antidepressant type, and IPT format.

Several limitations deserve mention. First, the number of studies in various secondary and sensitivity analyses was relatively small. Second, apart from one study (Elkin et al., 1989), data on what (if any) non-study treatment(s) patients received during follow-up were unavailable, limiting our ability to control for this important potential confound to longer-term follow-up. Third, the included studies' risk-of-bias scores were either high or of concern. It is worth noting that the Cochrane Collaboration's risk-of-bias tool for randomized trials was originally developed for conventional meta-analyses, and there is currently no adaptation specifically tailored for IPD meta-analyses (Higgins et al., 2022), while a high risk-of-bias score in certain domains is less problematic in the context of an IPD meta-analysis. For example, studies are deemed at risk-of-bias if publications do not report outcomes for all measures (i.e. selective reporting) or do not report outcomes for the full randomized sample (i.e. missing outcome data). These issues have less relevance for the current analyses, as IPD were requested for all outcome measures assessed, and for all patients randomized. In fact, intention-to-treat samples were available for most of the included studies (7/9 for the primary outcome), and for studies that did not retain data for all patients randomized, only a few patients (*N* = 34) were missing. Although adding risk-of-bias items as covariates did not change the direction or significance of the results, the limited variability in risk-of-bias item ratings indicates this finding requires cautious interpretation. Fourth, IPD were not obtained for about 20% of the eligible patient sample, with some indication of data-availability bias, as studies from the USA were less likely to contribute data. However, effect sizes did not differ significantly between studies for which IPD were and were not available, suggesting limited influence of data-availability bias on the effect estimates. Fifth, although many similarities were observed across study protocols, methodological differences did emerge in key areas, such as treatment length. While heterogeneity was generally low, *I*^2^ estimates need to be interpreted with caution in the analyses based on *z*-scores, as the standardization of outcomes might have reduced between-study variability. Sixth, all studies were conducted in Europe, Canada, and the USA, threatening the generalizability of findings to other regions.

### Clinical implications and future directions

Both IPT and antidepressants are recommended interventions for major depression and widely used across clinical settings. IPT's specific focus on current salient relational and interpersonal problems provides an important alternative to other psychotherapeutic approaches like CBT and psychodynamic therapy. This IPD meta-analysis indicates that individuals suffering from depression and their clinicians might expect similar improvements in depression, social functioning, quality of life, and coping after IPT and antidepressants, while antidepressants might result in somewhat lower levels of general psychopathology and dysfunctional attitudes than IPT at the end of acute treatment.

Given the study limitations, it is recommended that standard practice for future RCTs include multiple outcome measures, longer-term follow-up, and tracking of non-study treatment during the follow-up period. Considering the long-term nature of depression and potential advantages of IPT over antidepressants after acute treatment is completed (Imel et al., 2008), the scarcity of follow-up assessments is a true limitation to the current state of the literature. The field would highly benefit from longitudinal treatment comparisons in large-scale pragmatic trials. This will require increased support from funding agencies to offset study costs related to longer-term follow-up. The field would also benefit from consistent pre-registration of trial protocols and the availability of (open access) datasets for further study. Moreover, tools for evaluating the risk-of-bias in primary studies should be adapted for use in IPD meta-analyses, as the inability of existing evaluation frameworks to account for the analytic flexibility afforded by IPD meta-analyses' access to source data inflates bias estimates. The high rates of non-response (National Health Service, 2022; Papakostas & Fava, 2010; Rush et al., 2006) to existing treatments for depression highlights the importance of enhancing our understanding of what works for whom in order to match individuals to optimal treatments (Cohen & DeRubeis, 2018).

## Supporting information

Cohen et al. supplementary material Cohen et al. supplementary material

## Supplementary material

The supplementary material for this article can be found at https://doi.org/10.1017/S0033291724001788
