# Supplementary material for: Comparative efficacy of interpersonal psychotherapy and antidepressant medication for adult depression: a systematic review and individual participant data meta-analysis
Source: Psychol Med. 2024 Nov 4;54(14):3785–94. doi: 10.1017/S0033291724001788 (PMC11578913; doi:10.1017/S0033291724001788)
Supplement: Cohen et al. supplementary material [file S0033291724001788sup001.docx]

**Appendix**

| **Supporting information** | |
| --- | --- |
| *Divergences from the Protocol Article*  Analyses precluded by unavailable data: | |
|  | Sensitivity analysis examining the effect of studies that enrolled participants meeting diagnostic criteria for depression versus studies that enrolled participants presenting an elevated score on a standardized measure of depression. |
|  | Sensitivity analyses to control for the effects of additional non-study treatment during the trial and in the follow-up period by adding this data as covariate(s) to the models. |
|  | Sensitivity analyses only including studies that scored negative on all risk of bias items. |
|  | Data availability bias regarding effect sizes (Cohen’s *d*) of secondary outcome measures extracted from publications. |
|  | |

| **Table 1**  *Overview of Outcome Measures Used by the Included Studies* | | | | | | | | | | |
| --- | --- | --- | --- | --- | --- | --- | --- | --- | --- | --- |
| Construct | Measure | Altamura et al., 2017 | Blom et al., 2007 | Browne et al., 2002 | Elkin et al., 1989 | Finkenzeller et al., 2009 | Frank et al., 2011^a^ | Gois et al., 2014^a^ | Menchetti et al., 2014 | Quilty et al., 2008 |
| Depression | HAM-D-17 |  | x |  | x | x | x |  |  | x |
|  | HAM-D-21 | x |  |  |  |  |  |  | x |  |
|  | MADRS |  |  | x |  |  |  | x |  |  |
| Anxiety | HAM-A | x |  |  |  |  |  |  |  |  |
|  | HADS-A |  |  |  |  | x |  |  |  |  |
| General psychopathology | HSCL-90 |  |  |  | x |  |  |  |  |  |
|  | CGI |  | x |  |  |  |  |  |  |  |
|  | GAF |  |  |  |  |  |  |  |  | x |
| Quality of life | WHOQoL-BREF |  |  |  |  |  |  |  | x |  |
|  | Q-LES-Q |  |  |  |  |  | x |  |  |  |
| Social functioning | WSAS |  |  |  |  |  | x |  | x |  |
|  | SAS |  |  | x | x |  |  |  |  |  |
|  | SDS |  | x |  |  |  |  |  |  |  |
| Interpersonal problems | IIP-32 |  |  |  |  |  |  |  |  | x |
|  | IPS |  | x |  |  |  |  |  |  |  |
| Coping mechanisms | Problem solving |  | x^b^ | x^c^ |  |  |  |  |  |  |
|  | Avoidance |  | x^b^ | x^c^ |  |  |  |  |  |  |
| Personality traits | NEO-FFI |  | x |  |  |  |  |  |  | x |
| Dysfunctional attitudes | DAS |  |  |  | x |  |  |  |  | x |
| *Note.* CGI = Clinical Global Impression; DAS = Dysfunctional Attitudes Scale; GAF = Global Assessment of Functioning; HADS-A = Hospital Anxiety and Depression Scale, anxiety subscale; HAM-A = Hamilton Anxiety Rating Scale; HAM-D = Hamilton Depression rating scale, 17 and 21 items; HSCL-90 = 90-item Hopkins Symptom Checklist; IIP-32 = 32-item Inventory of Interpersonal Problems; IPS = Interpersonal Sensitivity; MADRS = Montgomery Åsberg Depression Rating Scale; NEO-FFI = NEO Five-Factor Inventory; Q-LES-Q = Quality of Life Enjoyment and Satisfaction Questionnaire; SAS = Social Adjustment Scale; SDS = Sheehan Disability Scale; WHOQoL-BREF = World Health Organization Quality of Life Scale-BREF; WSAS = Work and Social Adjustment Scale.  ^a^ Outcomes were based on data from 6 weeks post-baseline, as non-responding participants received augmentation with the other treatment after this point.  ^b^ From the Utrechtse Copinglijst (UCL)  ^c^ Coping mechanism domains were individually measured by the study authors and were not included in a specific instrument or questionnaire | | | | | | | | | | |

| **Table 2**  *References of the included studies* | |
| --- | --- |
| Study | Reference |
| Individual participant data available | |
| Altamura et al., 2017 | Altamura M, Iuso S, Terrone G, Balzotti A, Carnevale R, Malerba S, et al. Comparing interpersonal counseling and antidepressant treatment in primary care patients with anxious and nonanxious major depression disorder: a randomized control trial. Clin Neuropsychiatry. 2017; 14(4). |
| Blom et al., 2007 | Blom MB, Jonker K, Dusseldorp E, Spinhoven P, Hoencamp E, Haffmans J, et al. Combination treatment for acute depression is superior only when psychotherapy is added to medication. Psychother and Psychosom. 2007; 76(5): 289-97. |
| Browne et al., 2002 | Browne G, Steiner M, Roberts J, Gafni A, Byrne C, Dunn E, et al. Sertraline and/or interpersonal psychotherapy for patients with dysthymic disorder in primary care: 6-month comparison with longitudinal 2-year follow-up of effectiveness and costs. J Affect Disord. 2002; 68(2-3): 317-30. |
| Elkin et al., 1989 | Elkin I, Shea MT, Watkins JT, Imber SD, Sotsky SM, Collins JF, et al. National Institute of Mental Health treatment of depression collaborative research program: General effectiveness of treatments. Arch Gen Psychiatry. 1989; 46(11): 971-82. |
| Finkenzeller et al., 2009 | Finkenzeller W, Zobel I, Rietz S, Schramm E, Berger M. Interpersonal psychotherapy and pharmacotherapy for post-stroke depression. Feasibility and effectiveness. Nervenarzt. 2009; 80(7): 805-12. |
| Frank et al., 2011 | Frank E, Cassano GB, Rucci P, Thompson WK, Kraemer HC, Fagiolini A, et al. Predictors and moderators of time to remission of major depression with interpersonal psychotherapy and SSRI pharmacotherapy. Psychol Med. 2011; 41(1): 151-62. |
| Gois et al., 2014 | Gois C, Dias V, Carmo I, Duarte R, Ferro A, Santos A, et al. Treatment response in type 2 diabetes patients with major depression. Clin Psychol and Psychother. 2014; 21(1): 39-48. |
| Menchetti et al., 2014 | Menchetti M, Rucci P, Bortolotti B, Bombi A, Scocco P, Kraemer HC, et al. Moderators of remission with interpersonal counselling or drug treatment in primary care patients with depression: randomised controlled trial. Br J Psychiatry. 2014; 204(2): 144-50. |
| Quilty et al., 2008 | Quilty L, McBride C, Bagby R. Evidence for the cognitive mediational model of cognitive behavioural therapy for depression. Psychol Med. 2008; 38(11): 1531-41. |
| No individual participant data available | |
| Markowitz et al., 2005 | Markowitz JC, Kocsis JH, Bleiberg KL, Christos PJ, Sacks M. A comparative trial of psychotherapy and pharmacotherapy for “pure” dysthymic patients. J Affect Disord. 2005; 89(1-3): 167-75. |
| Martin et al., 2001 | Martin SD, Martin E, Rai SS, Richardson MA, Royall R. Brain blood flow changes in depressed patients treated with interpersonal psychotherapy or venlafaxine hydrochloride: preliminary findings. Arch Gen Psychiatry. 2001; 58(7): 641-8. |
| O’Hara et al., 2019 | O'Hara MW, Pearlstein T, Stuart S, Long JD, Mills JA, Zlotnick C. A placebo controlled treatment trial of sertraline and interpersonal psychotherapy for postpartum depression. J Affect Disord. 2019; 245: 524-32. |
| Schulberg et al., 1996 | Schulberg HC, Block MR, Madonia MJ, Scott CP, Rodriguez E, Imber SD, et al. Treating major depression in primary care practice: eight-month clinical outcomes. Arch Gen Psychiatry. 1996; 53(10): 913-9. |
| Sloane et al., 1985 | Sloane BR, Staples FR, Schneider LS. Interpersonal therapy versus nortriptyline for depression in the elderly. Clinical and Pharmacological Studies in Psychiatric Disorders. 1985: 344-6. |
| Weissman et al., 1979 | Weissman MM, Prusoff BA, Dimascio A, Neu C, Goklaney M, Klerman GL. The efficacy of drugs and psychotherapy in the treatment of acute depressive episodes. Am J Psychiatry. 1979; 136(4B): 555-8. |

| **Table 3**  *Comparison of Study Characteristics based on IPD Availability* | | | | | |
| --- | --- | --- | --- | --- | --- |
| Variable | Individual participant data | | χ² | df | *p* |
|  | Available | Unavailable |  |  |  |
| Recruitment |  |  | 2.508 | 3 | .474 |
| Community | 0 | 1 |  |  |  |
| Clinical | 4 | 2 |  |  |  |
| Other | 2 | 2 |  |  |  |
| Mixed | 3 | 1 |  |  |  |
| Country |  |  | 9.377 | 3 | .025* |
| Europe | 5 | 1 |  |  |  |
| Canada | 2 | 0 |  |  |  |
| USA | 1 | 5 |  |  |  |
| Mixed | 1 | 0 |  |  |  |
| Target group |  |  | 5.770 | 3 | .123 |
| Adults | 7 | 4 |  |  |  |
| Older adults (mean age > 55) | 0 | 1 |  |  |  |
| Women with Postpartum depression | 0 | 1 |  |  |  |
| General medical disorder | 2 | 0 |  |  |  |
| Depression inclusion criteria |  |  | 2.246 | 1 | .134 |
| Clinicians’ diagnostic criteria | 2 | 0 |  |  |  |
| Elevated depression score | 0 | 0 |  |  |  |
| Mixed | 7 | 6 |  |  |  |
| * Statistical significance (*p* < 0.05). | | | | | |

| **Table 4** | | | | | | |
| --- | --- | --- | --- | --- | --- | --- |
| *Sensitivity Analyses of the Comparative Treatment Effects of IPT and Antidepressants at Post-Treatment and Follow-Up* | | | | | | |
|  |  |  |  | 95% CI | |  |
| Outcome^a^ | *k* | *N* | *d*^b^ | Lower | Upper | *p* |
| Post-treatment | | | | | | |
| Depression | 9 | 1530 | 0.088 | -0.018 | 0.194 | .103 |
| Unstandardised HAM-D-17 scores | 5 | 695 | 0.650 | -0.220 | 1.520 | .143 |
| Unstandardised HAM-D-21 scores | 2 | 341 | -0.798 | -1.711 | 0.115 | .087 |
| Unstandardised MADRS scores | 2 | 494 | 2.413 | 0.835 | 3.991 | .003* |
| RoB items as coviarates | 9 | 1530 | 0.089 | -0.017 | 0.195 | .099 |
| Treatment quality items as covariates | 9 | 1530 | 0.089 | -0.017 | 0.195 | .099 |
| High treatment quality studies only | 5 | 1260 | 0.139 | 0.021 | 0.257 | .021* |
| Studies using IPT only | 7 | 1189 | 0.170 | 0.048 | 0.292 | .006* |
| Studies using IPC only | 2 | 341 | -0.173 | -0.383 | 0.037 | .106 |
| Studies with incl. criteria for dysthymia only | 1 | 460 | 0.256 | 0.056 | 0.456 | .012* |
| Studies with incl. criteria for unipolar mood disorder only | 8 | 1070 | 0.021 | -0.104 | 0.146 | .743 |
| Primary care studies only | 3 | 801 | 0.061 | -0.084 | 0.206 | .410 |
| Secondary or tertiary care studies only | 6 | 729 | 0.119 | -0.036 | 0.274 | .132 |
| Studies including adults only | 8 | 1479 | 0.095 | -0.013 | 0.203 | .084 |
| Studies including older adults only | 1 | 51 | -0.084 | -0.570 | 0.402 | .735 |
| Studies using SSRI only | 6 | 1177 | 0.075 | -0.043 | 0.193 | .211 |
| Studies using SARI only | 1 | 97 | -0.060 | -0.534 | 0.414 | .804 |
| Studies using TCA only | 1 | 126 | 0.544 | 0.127 | 0.961 | .011* |
| Studies with post-treatment at 4-8 weeks only | 5 | 717 | -0.028 | -0.173 | 0.117 | .705 |
| Studies with post-treatment at 12-20 weeks only | 3 | 353 | 0.141 | -0.094 | 0.376 | .240 |
| Studies with post-treatment at 26 weeks only | 1 | 460 | 0.256 | 0.056 | 0.456 | .012* |
| Social functioning | 5 | 1213 | 0.026 | -0.078 | 0.130 | .624 |
| Unstandardized WSAS scores | 2 | 567 | 0.461 | -0.866 | 1.788 | .496 |
| RoB items as coviarates | 5 | 1213 | 0.026 | -0.078 | 0.130 | .624 |
| Treatment quality items as covariates | 5 | 1213 | 0.026 | -0.078 | 0.130 | .624 |
| High treatment quality studies only | 5 | 1213 | 0.026 | -0.078 | 0.130 | .624 |
| Studies using IPT only | 4 | 934 | 0.046 | -0.074 | 0.166 | .451 |
| Studies using IPC only | 1 | 279 | -0.028 | -0.240 | 0.184 | .795 |
| Studies with incl. criteria for dysthymia only | 1 | 425 | -0.002 | -0.174 | 0.170 | .982 |
| Studies with incl. criteria for unipolar mood disorder only | 4 | 788 | 0.046 | -0.083 | 0.175 | .486 |
| Primary care studies only | 2 | 704 | 0.021 | -0.114 | 0.156 | .761 |
| Secondary or tertiary care studies only | 3 | 509 | 0.088 | -0.075 | 0.251 | .289 |
| Studies using SSRI only | 3 | 992 | 0.022 | -0.092 | 0.136 | .705 |
| Studies using SARI only | 1 | 96 | -0.110 | -0.451 | 0.231 | .527 |
| Studies using TCA only | 1 | 125 | 0.219 | -0.163 | 0.601 | .261 |
| Studies with post-treatment at 4-8 weeks only | 2 | 567 | 0.052 | -0.097 | 0.201 | .494 |
| Studies with post-treatment at 12-20 weeks only | 2 | 221 | 0.040 | -0.215 | 0.295 | .758 |
| Studies with post-treatment at 26 weeks only | 1 | 425 | -0.002 | -0.174 | 0.170 | .982 |
| General psychopathology | 3 | 307 | 0.276 | 0.039 | 0.513 | .023* |
| RoB items as covariates | 3 | 307 | 0.277 | 0.040 | 0.514 | .022* |
| Treatment quality items as covariates | 3 | 307 | 0.277 | 0.040 | 0.514 | .022* |
| High treatment quality studies only | 2 | 202 | 0.122 | -0.164 | 0.408 | .403 |
| Studies using SARI only | 1 | 81 | -0.168 | -0.576 | 0.240 | .419 |
| Studies using TCA only | 1 | 121 | 0.396 | 0.000 | 0.792 | .050* |
| Quality of Life | 2 | 567 | -0.049 | -0.198 | 0.100 | .519 |
| RoB items as covariates | 2 | 567 | -0.049 | -0.198 | 0.100 | .519 |
| Treatment quality items as covariates | 2 | 567 | -0.049 | -0.198 | 0.100 | .519 |
| High treatment quality studies only | 2 | 567 | -0.049 | -0.198 | 0.100 | .519 |
| Studies using IPT only | 1 | 286 | -0.172 | -0.378 | 0.034 | .101 |
| Studies using IPC only | 1 | 281 | 0.066 | -0.148 | 0.280 | .545 |
| Primary care studies only | 1 | 281 | 0.066 | -0.148 | 0.280 | .545 |
| Secondary or tertiary care studies only | 1 | 286 | -0.172 | -0.378 | 0.034 | .101 |
| Coping – Problem solving | 2 | 513 | -0.059 | -0.226 | 0.108 | .488 |
| RoB items as covariates | 2 | 513 | -0.059 | -0.226 | 0.108 | .488 |
| Treatment quality items as covariates | 2 | 513 | -0.059 | -0.226 | 0.108 | .488 |
| High treatment quality studies only | 2 | 513 | -0.059 | -0.226 | 0.108 | .488 |
| Studies with incl. criteria for dysthymia only | 1 | 425 | -0.080 | -0.272 | 0.112 | .414 |
| Studies with incl. criteria for unipolar mood disorder only | 1 | 88 | 0.038 | -0.260 | 0.336 | .803 |
| Primary care studies only | 1 | 425 | -0.080 | -0.272 | 0.112 | .414 |
| Secondary or tertiary care studies only | 1 | 88 | 0.038 | -0.260 | 0.336 | .803 |
| Studies using SSRI only | 1 | 425 | -0.080 | -0.272 | 0.112 | .414 |
| Studies using SARI only | 1 | 88 | 0.038 | -0.260 | 0.336 | .803 |
| Studies with post-treatment at 12-20 weeks only | 1 | 88 | 0.038 | -0.260 | 0.336 | .803 |
| Studies with post-treatment at 26 weeks only | 1 | 425 | -0.080 | -0.272 | 0.112 | .414 |
| Coping - Avoidance | 2 | 509 | 0.041 | -0.118 | 0.200 | .613 |
| RoB items as covariates | 2 | 509 | 0.041 | -0.118 | 0.200 | .613 |
| Treatment quality items as covariates | 2 | 509 | 0.041 | -0.118 | 0.200 | .613 |
| High treatment quality studies only | 2 | 509 | 0.041 | -0.118 | 0.200 | .613 |
| Studies with incl. criteria for dysthymia only | 1 | 425 | 0.079 | -0.099 | 0.257 | .385 |
| Studies with incl. criteria for unipolar mood disorder only | 1 | 84 | -0.179 | -0.500 | 0.142 | .275 |
| Primary care studies only | 1 | 425 | 0.079 | -0.099 | 0.257 | .385 |
| Secondary or tertiary care studies only | 1 | 84 | -0.179 | -0.500 | 0.142 | .275 |
| Studies using SSRI only | 1 | 425 | 0.079 | -0.099 | 0.257 | .385 |
| Studies using SARI only | 1 | 84 | -0.179 | -0.500 | 0.142 | .275 |
| Studies with post-treatment at 12-20 weeks only | 1 | 84 | -0.179 | -0.500 | 0.142 | .275 |
| Studies with post-treatment at 26 weeks only | 1 | 425 | 0.079 | -0.099 | 0.257 | .385 |
| Dysfunctional Attitudes | 2 | 231 | 0.249 | 0.026 | 0.472 | .029* |
| Unstandardized DAS scores | 2 | 231 | 8.550 | 1.110 | 15.990 | .024* |
| RoB items as covariates | 2 | 231 | 0.249 | 0.026 | 0.472 | .029* |
| Treatment quality items as covariates | 2 | 231 | 0.249 | 0.026 | 0.472 | .029* |
| High treatment quality studies only | 1 | 122 | 0.130 | -0.180 | 0.440 | .411 |
| Personality – Neuroticism | 2 | 192 | 0.102 | -0.188 | 0.392 | .491 |
| RoB items as covariates | 2 | 192 | 0.101 | -0.189 | 0.391 | .495 |
| Treatment quality items as covariates | 2 | 192 | 0.101 | -0.189 | 0.391 | .495 |
| High treatment quality studies only | 1 | 87 | 0.171 | -0.315 | 0.657 | .490 |
| Personality – Extraversion | 2 | 192 | -0.054 | -0.293 | 0.185 | .658 |
| RoB items as covariates | 2 | 192 | -0.054 | -0.293 | 0.185 | .658 |
| Treatment quality items as covariates | 2 | 192 | -0.054 | -0.293 | 0.185 | .658 |
| High treatment quality studies only | 1 | 87 | -0.226 | -0.632 | 0.180 | .275 |
| Personality – Agreeableness | 2 | 192 | 0.169 | -0.113 | 0.451 | .241 |
| RoB items as covariates | 2 | 192 | 0.168 | -0.114 | 0.450 | .243 |
| Treatment quality items as covariates | 2 | 192 | 0.168 | -0.114 | 0.450 | .243 |
| High treatment quality studies only | 1 | 87 | 0.441 | -0.033 | 0.915 | .068 |
| Personality – Conscientiousness | 2 | 192 | -0.050 | -0.297 | 0.197 | .691 |
| RoB items as covariates | 2 | 192 | -0.050 | -0.297 | 0.197 | .691 |
| Treatment quality items as covariates | 2 | 192 | -0.050 | -0.297 | 0.197 | .691 |
| High treatment quality studies only | 1 | 87 | -0.099 | -0.542 | 0.344 | .661 |
| Personality – Openness | 2 | 191 | 0.133 | -0.110 | 0.376 | .283 |
| RoB items as covariates | 2 | 191 | 0.133 | -0.110 | 0.376 | .283 |
| Treatment quality items as covariates | 2 | 191 | 0.133 | -0.110 | 0.376 | .283 |
| High treatment quality studies only | 1 | 86 | 0.106 | -0.337 | 0.549 | .640 |
| Interpersonal problems | 2 | 190 | -0.038 | -0.263 | 0.187 | .741 |
| RoB items as covariates | 2 | 190 | -0.038 | -0.263 | 0.187 | .741 |
| Treatment quality items as covariates | 2 | 190 | -0.038 | -0.263 | 0.187 | .741 |
| High treatment quality studies only | 1 | 86 | -0.031 | -0.323 | 0.261 | .835 |
| Anxiety | 2 | 106 | -0.200 | -0.555 | 0.155 | .269 |
| RoB items as covariates | 2 | 106 | -0.201 | -0.558 | 0.156 | .269 |
| Treatment quality items as covariates | 2 | 106 | -0.201 | -0.558 | 0.156 | .269 |
| Studies using IPT only | 1 | 51 | 0.073 | -0.423 | 0.569 | .773 |
| Studies using IPC only | 1 | 55 | -0.531 | -1.041 | -0.021 | .041* |
| Primary care studies only | 1 | 55 | -0.531 | -1.041 | -0.021 | .041* |
| Secondary or tertiary care studies only | 1 | 51 | 0.073 | -0.423 | 0.569 | .773 |
| Studies including adults only | 1 | 55 | -0.531 | -1.041 | -0.021 | .041* |
| Studies including older adults only | 1 | 51 | 0.073 | -0.423 | 0.569 | .773 |
| Follow-up | | | | | | |
| Depression | 3 | 716 | 0.150 | -0.023 | 0.323 | .088 |
| RoB items as covariates | 3 | 716 | 0.149 | -0.025 | 0.322 | .090 |
| Treatment quality items as covariates | 3 | 716 | 0.149 | -0.025 | 0.322 | .090 |
| One year follow-up period | 2 | 586 | 0.134 | -0.046 | 0.314 | .145 |
| Primary care studies only | 1 | 460 | 0.275 | 0.067 | 0.483 | .009* |
| Secondary or tertiary care studies only | 2 | 256 | -0.116 | -0.430 | 0.198 | .468 |
| Studies using SSRI only | 1 | 460 | 0.275 | 0.067 | 0.483 | .009* |
| Studies using TCA only | 1 | 126 | -0.349 | -0.743 | 0.045 | .082 |
| Studies with incl. criteria for dysthymia only | 1 | 460 | 0.275 | 0.067 | 0.483 | .009* |
| Studies with incl. criteria for unipolar mood disorder only | 2 | 256 | -0.116 | -0.743 | 0.045 | .468 |
| Social functioning | 2 | 550 | -0.014 | -0.179 | 0.151 | .868 |
| RoB items as covariates | 2 | 550 | -0.014 | -0.179 | 0.151 | .868 |
| Treatment quality items as covariates | 2 | 550 | -0.014 | -0.179 | 0.151 | .868 |
| One year follow-up period | 2 | 550 | -0.072 | -0.229 | 0.085 | .368 |
| Primary care studies only | 1 | 425 | 0.117 | -0.067 | 0.301 | .213 |
| Secondary or tertiary care studies only | 1 | 125 | -0.477 | -0.836 | -0.118 | .009* |
| Studies using SSRI only | 1 | 425 | 0.117 | -0.067 | 0.301 | .213 |
| Studies using TCA only | 1 | 125 | -0.477 | -0.836 | -0.118 | .009* |
| Studies with incl. criteria for dysthymia only | 1 | 425 | 0.117 | -0.067 | 0.301 | .213 |
| Studies with incl. criteria for unipolar mood disorder only | 1 | 125 | -0.477 | -0.836 | -0.118 | .009* |
| General Psychopathology | 2 | 226 | -0.003 | -0.309 | 0.303 | .985 |
| RoB items as covariates | 2 | 226 | -0.003 | -0.309 | 0.303 | .985 |
| Treatment quality items as covariates | 2 | 226 | -0.003 | -0.309 | 0.303 | .985 |
| High treatment quality studies only | 1 | 121 | -0.299 | -0.664 | 0.066 | .108 |
| *Note*. *k =* number of studies; *N* = number of patients; *d =* Cohen’s *d* effect size; DAS = Dysfunctional Attitudes Scale; HAM-D = Hamilton Depression Rating Scale; IPC = Interpersonal Counseling; IPT = Interpersonal Psychotherapy; MADRS = Montgomery Åsberg Depression Rating Scale; RoB = Risk of Bias; SARI = Serotonin Antagonist and Reuptake Inhibitor; SSRI = Selective Serotonin Reuptake Inhibitor; TCA = Tricyclic Antidepressant; WSAS = Work and Social Adjustment Scale.  ^a^ Studies measuring interpersonal problems, personality traits, and dysfunctional attitudes at post-treatment had similar study characteristics, and therefore it was not able to make subgroup comparisons. This also applied for studies measuring general psychopathology at follow-up.  ^b^ Positive effect sizes indicate an advantage of antidepressants over IPT, except for the quality of life outcome measure where a negative effect size indicates a favourability of antidepressants over IPT. | | | | | | |
